# The impact of the digital economy on high-quality agricultural development——Based on the regulatory effects of financial development

**DOI:** 10.1371/journal.pone.0293538

**Published:** 2024-03-06

**Authors:** Li Zhou, Shuning Zhang, Chenjing Zhou, Shuai Yuan, Hong Jiang, Yifeng Wang

**Affiliations:** 1 Suqian University, Suqian, China; 2 City University of Macau, Macau, China; 3 Lanzhou University of Finance and Economics, Lanzhou, China; 4 Suqian Municipal Government Office, Suqian, China; Sichuan University, CHINA

## Abstract

The digital economy, as a new economic form with high information density, provides a new driving force for the realization of high-quality agricultural development. Panel data of 31 provinces in China from 2011 to 2020 were selected for analysis. The static panel data interaction effect model and panel threshold model were used to verify the nonlinear influence mechanism and heterogeneity of financial development in the process of the digital economy affecting high-quality agricultural development. The findings are as follows. (1) During the study period, the high-quality development of China’s agriculture showed a steady upward trend; however, the regional differences were significant, and the eastern part was larger than the central and western part. (2) The digital economy can promote high-quality agricultural development. (3) The digital economy has a double threshold effect in the process of affecting high-quality agricultural development, which depends on the level of financial development. When the threshold is exceeded, the digital economy has a more significant promoting effect on high-quality agricultural development. (4) The impact of the digital economy on high-quality agricultural development is heterogeneous. From the perspective of different regions, the impact effect is greatest in the eastern region, while the effect is smaller in the central and western regions. From different resource endowments, the positive impact effect is greatest in the major grain-selling areas, followed by the major grain producing areas, but the positive digital economy driving effect is not significant in the balance of production and sales areas. Finally, three policy suggestions are proposed. First, the Chinese government should increase investments in and support for digital technology to promote the integration of the digital economy and agriculture. Second, the Chinese government should promote the development of digital inclusive finance in areas with financial development below the threshold. Third, different regions should implement differentiated digital economies to promote high-quality agricultural development.

## Introduction

As China is a large agricultural country, emphasizing agriculture and consolidating the basic is the basis for the country’s security and governance. Achieving high-quality agricultural development is the foundation and key to China’s economic development. It is also an important measure to better meet people’s aspirations for a better life. In recent years, China’s overall agricultural production capacity has increased significantly, providing the material foundation and social conditions for the transformation of agricultural development from increasing production to improving agricultural quality. However, given the background of a series of objective realities, such as more people and less land, land fragmentation and small farmers’ dispersion, the contradiction between the traditional agricultural production mode and the realization of high-quality agricultural development has become increasingly apparent. Agricultural production efficiency is low, industrial competitiveness is not strong, production fragmentation is relatively high, and continually increasing farmers’ incomes is more difficult. As a long-lasting and complex systematic project, not only does the realization of high-quality agricultural development face prominent bottlenecks but also traditional agricultural production technology has been unable to provide a sustainable driving force for the realization of high-quality agricultural development. The rapid development of the digital economy provides a historic opportunity to solve this problem. The digital economy represents a new technological change and a new development power that inevitably has a substantial impact on the development of traditional agriculture at all levels, injecting "digital power" into the promotion of agricultural transformation and upgrading, promoting the sustainable development of agriculture, and changing the agricultural production, operational and management modes. The digital economy has deepened the division of labor, extended the agricultural industrial and value chains, saved transaction costs, and improved agricultural production efficiency and development quality. Therefore, the development of the digital economy has become the focus and new engine to promote the development of high-quality agriculture. Studying the impact of the digital economy on the development of high-quality agriculture and its action path has important research value.

At this stage, a large number of scholars in the academic community have studied the impact of the digital economy on farmers, agriculture and rural development from different perspectives. Theoretically, they agree that the digital economy can promote high-quality development of agriculture, but there is a lack of specific empirical research support. In addition, the existing literature has not yet studied the regulatory mechanism of financial development in the digital economy affecting high-quality agricultural development.

The innovations are mainly reflected in the following aspects. (1) A comprehensive evaluation index system of high-quality agricultural development was constructed from five dimensions: innovation, coordination, green, openness and sharing. The entropy method was used to measure the development of China’s high-quality agriculture. (2) A kernel density estimation was used to analyze the dynamic evolution characteristics of high-quality agricultural development in China and to effectively grasp its law of evolution given changes in time and space. (3) The static panel data interaction effect model and panel threshold model were used to analyze the nonlinear influence mechanism of financial development in the process of the digital economy affecting high-quality agricultural development.

Compared with the existing literature, the possible marginal contributions of this paper lie in the following three aspects. Firstly, most of the existing literature studies the impact of the Internet on agricultural digitalization, agricultural structural adjustment and rural economy from a single perspective, while there are few empirical studies on the comprehensive impact of the digital economy on the high-quality development of agriculture. This paper connects digital economy with agricultural development, uses empirical analysis to explore the impact of digital economy on high-quality agricultural development, and verifies the theoretical research conclusions of previous literature on the positive driving effect of digital economy on high-quality agricultural development. Secondly, the heterogeneity of the impact of digital economy on agricultural high-quality development is explored from the dimensions of geographic location and resource endowment, which further enriches the research results on the relationship between digital economy and agricultural high-quality development. Thirdly, different from previous literatures, this paper examines the threshold effect of financial development in the impact of digital economy on high-quality agricultural development, and focuses on the impact of this effect on high-quality agricultural development. This conclusion further clarifies that improving financial development level is the key point for local governments to promote the positive driving effect of digital economy on high-quality agricultural development.

## Literature review

Among the existing research results, there are few special studies on the relationship between the digital economy and high-quality agricultural development, but there are many related studies.

### Related research on the digital economy

Research on the digital economy mainly focuses on its economic effect, including the following three aspects.

First, the impact of the digital economy on high-quality development. Ding CH (2022) noted that the digital economy can promote the high-quality development of China’s economy, and technological innovation is an important channel for the digital economy to develop into a high-quality economy [1]. Xu S (2022) analyzed and measured the relationship between the digital economy and environmental pollution in 287 prefecture-level cities in China from 2008 to 2018 by using a simultaneous spatial equation and the generalized three-stage least squares (GS3SLS) method and noted that the digital economy is of great significance in achieving green and high-quality development [2].

Second, the impact of the digital economy on energy consumption. Shahbaz M (2022) used panel data from 72 countries from 2003 to 2019 to study the impact of the digital economy on renewable energy consumption and the power generation structure and found that the digital economy has a positive impact on energy transition [3]. Xue Y (2022) noted that the development of the digital economy expands energy consumption and the optimization of the energy consumption structure [4].

Third, the impact of the digital economy on green development. Li ZG (2022) found that the digital economy is of great significance to carbon emissions reductions, which is necessary to strengthen the digital economy and promote regional environmental governance cooperation to achieve carbon peak and neutrality [5]. Zhang JN (2022) constructed a digital economy evaluation system from four dimensions, namely, the development carrier of the digital economy, digital industrialization, industrial digitalization, and the development environment of the digital economy, and conducted an analysis using the intermediary effect model and found that the digital economy is gradually becoming an important driving force for regional low-carbon development [6]. Wang LL (2022) noted that vigorously developing the digital economy can effectively promote the low-carbon and sustainable development of cities [7]. Zhang W (2022) found that the digital economy improves carbon emission performance; however, given different energy consumption structures, ecological environments, government interventions and urban greening levels, the influence of the ecological environment on climate change is nonlinear [8]. Li JL (2022) built a digital economy index and green economy efficiency index based on the panel data of 277 cities in China from 2011 to 2018 and noted that the digital economy significantly improved the efficiency of the regional green economy and that technological innovation was an important way for the digital economy to improve the efficiency of the green economy [9]. Liu Y (2022) studied the relationship among digital economy development, industrial structure upgrading and green total factor productivity (GTFP) by using Tobit, quantile regression, impulse response function and mediation effect models. The results showed that the digital economy has a positive promoting effect on China’s GTFP. In addition, industrial structure upgrading is the intermediary transmission mechanism of the digital economy promoting GTFP [10]. Pan WR (2022) found that the digital economy has an innovation-driven effect on the extensive and sustainable development of total factor productivity; however, the promoting effect of digital integration on the high-quality growth of total factor productivity is different in different regions, while the promoting effect on the central and western regions is relatively weak [11]. DR Han (2022) noted that the digital economy can promote total factor carbon productivity in China and can become a new energy for the country to improve its green development [12]. Ren SM (2022) found that digital economy agglomeration affects inclusive green growth through energy consumption, environmental pollution, economic growth, human capital, industrial structure and technological progress [13].

### Related research on high-quality agricultural development

Research on high-quality agricultural development mainly focuses on the connotation of high-quality agricultural development and the measurement of high-quality agricultural levels.

#### Research on the connotation and measurement of high-quality agricultural development

At present, scholars have two views on the connotation of high agricultural quality. (1) The connotation of agricultural high-quality development is single, the quality of economic growth is equal to economic efficiency [14, 15], and the core of the quality of agricultural economic growth is agricultural efficiency [16]. (2) The connotation of high-quality agricultural development is multidimensional, systematic and dynamic. A single dimension is not sufficient to fully depict high-quality agricultural development. For example, Sun JC (2019) proposed that high-quality agricultural development should reflect the features of outstanding benefits, optimized structure and sustainable development and embody the development concept of "innovation, coordination, green, open and sharing" [17].

On the basis of the in-depth cognition of the connotation of high agricultural quality, many scholars have chosen to measure the development of high agricultural quality from a multidimensional perspective. Cui XF (2022), based on the new development concept of the Chinese government, constructed an evaluation framework of innovation-coordinated—green—open—sharing high-quality agricultural development and uses the system integration model to conduct a quantitative evaluation of the level of high-quality agricultural development of the Yangtze River Economic Belt in China. The research found that the high-quality agricultural development of the Yangtze River Economic Belt in general shows a fluctuating trend. However, there are regional differences [18]. Jiang GX (2022) explored and established an index system and measurement method for evaluating high-quality agricultural development on the basis of the original evaluation system of agricultural modernization. At the same time, combined with big data technology, he built a research model for measuring the high-quality development differences in provincial rural economies, built system modules, and analyzed the system process [19].

#### Study on influencing factors of high-quality agricultural development

Regarding the research on the influencing factors of high-quality agricultural development, the existing results mainly indicate the influence on high-quality agricultural development from the perspectives of agricultural ecological efficiency, agricultural total factor productivity and green agricultural production. Wang GF (2022) and Chi MJ (2022) noted that agroecological efficiency is the key link to high-quality agricultural development [20, 21]. Wu GY (2022) noted that pilot policies of low carbon trading, regional economic development, population growth, urbanization and urban innovation have a significant impact on efficiency and agricultural ecological efficiency, and improving agricultural ecological efficiency can promote high-quality agricultural development [22]. Qin S et al. (2022) analyzed the relationship between factor mismatches and high-quality agricultural development based on China’s interprovincial panel data from 2004 to 2020 and found that factor mismatches significantly inhibited improvements in high-quality agricultural development, and the inhibition effect had obvious spatial-temporal heterogeneity [23]. Du L et al. (2023) and XW (2023) noted that improving agricultural total factor productivity is the key to promoting high-quality agricultural development [24, 25]. Xing Deng HY (2022) noted that high-quality agricultural development requires not only the continuous growth of agricultural productivity but also green agricultural production [26]. Milosevic G (2020) found that countries can often use fiscal policy as a tool to regulate the relationship between production and trade, and the tax burden is crucial to achieving sustainable agricultural development [27]. Guo XM (2020) noted that the development of agricultural mechanization can promote high-quality agricultural development; however, agriculture facing substantial environmental pressure also needs to transform to green and sustainable development [28].

### Research on the relationship between the digital economy and high-quality agricultural development

There are only a few special studies on the relationship between the digital economy and high-quality agricultural development. Previous scholars have mainly studied the impact of the digital economy on rural households, agriculture and rural development from different perspectives. Mei Y (2022) empirically tested the direct and indirect impacts of rural digital construction on the high-quality development of the rural economy based on a fixed effect model and an intermediary effect model and found a positive correlation between digital village construction and the high-quality development of the rural economy. The digital industry’s entrepreneurial activities are an important mechanism for digital rural construction to promote the high-quality development of the rural economy [29]. Tang Y (2022) based on China’s provincial panel data from 2011 to 2020, studied the influence mechanism of agricultural digitalization on the agricultural field by adopting the bidirectional fixed effect model and threshold effect test model and noted that agricultural digitalization was conducive to promoting high-quality agricultural development [30]. Liu F (2022) proposed a data-driven evaluation method for regional agricultural production efficiency and found that technical efficiency was the core driving factor for improving regional agricultural production efficiency [31]. Savchenko IV (2019) discussed the development priorities of crop production in Russia and noted that high-yield and environmentally friendly crop cultivation relying on digital intelligent production technology is one of the country’s main development priorities [32]. Ju XX (2022) noted that big data technology can promote adjustments to the agricultural structure and the high-quality development of modern agriculture, and the development of modern agriculture cannot be separated from agricultural industrialization, agricultural globalization, agricultural digitalization, agricultural integration, agricultural structure adjustment and agricultural innovation [33]. Shen ZY (2022) studied the relationship between China’s agricultural digital transformation and green growth and found that China’s provincial agriculture has experienced significant green growth driven by technological progress, and the popularization of the internet and digital technology has indeed promoted sustainable agricultural development [34]. Dai XW (2023) noted that accelerating the integration of digital technology and agriculture is the key to promoting the high-quality development of China’s agriculture [35]. The results of Yao W (2023) show that the development of the digital economy has promoted the high-quality development of agriculture, and the promotion effect in the eastern region is stronger than that in the central and western regions [36].

In summary, the literature provides a good theoretical basis for understanding the connotation of the digital economy and high-quality agricultural development and their relationship; however, there are fewer studies using empirical methods to study their relationship, and few studies have analyzed the impact path of the digital economy on high-quality agricultural development from the perspective of financial development. In view of this, based on existing research results, in this paper, we discuss the theoretical mechanism of the impact of the digital economy on high-quality agricultural development, use empirical methods to test the impact of the digital economy on high-quality agricultural development, and further discuss how the regulation of financial development affects the impact of the digital economy on high-quality agricultural development.

## Theoretical analysis and hypothesis

### The digital economy’s theoretical effect on high-quality agricultural development

The realization of high-quality agricultural development is a complex and systematic process. As an important driving force for current economic growth, the digital economy is becoming the core factor and source of power of high-quality agricultural development and provides an important path for comprehensively improving its quality and efficiency. The digital economy applies big data, cloud computing, 5G and other information technologies to the agricultural field, provides digital technical support for the development of the agricultural industry, realizes the deep integration of the new generation of information technology with the agricultural industry, and provides it with an intelligent and digital production mode. Specifically, first, the precise matching effect. Digital technology enables farmers to quickly, effectively and accurately obtain market information, such as on supply, demand and prices, adjust the planting structure and sales strategy of agricultural products in real time, accurately match market demand, and reduce information asymmetry and mismatches between supply and demand and the ineffective supply caused by information lags. Second, the efficiency enhancement effect. The digital economy can improve industrial efficiency in all aspects. In the planting link, farmers can use digital technology to complete detailed operations at various links, such as rice transplanting, fertilization, water spraying and harvesting, which helps improve labor efficiency. In the production link, traceability management of agricultural products from farmland to table should be established. Once the quality problems of agricultural products occur, they can be quickly traced to the source, and emergency treatment can be applied. In the agricultural product sales link, digital platforms are used to build multiple online and offline sales channels to enable consumers to query and provide feedback in real time to improve transaction efficiency. Third, economies of scale. The development of digital technology, especially the internet, has changed the traditional transaction method. Convenient shopping channels stimulate the growth in consumption and expand product sales coverage. As a result, farmers and agribusinesses are able to scale up planting and production, achieving economies of scale. Based on the above analysis, we propose the following research hypothesis.

H1: The digital economy can effectively promote high-quality agricultural development.

### The effect of the regulation mechanism of financial development in the digital economy on high-quality agricultural development

The different levels of economic development, location conditions and policy orientations in different regions of China cause significant regional differences in financial development. Moreover, given continuous improvements in the openness of the market economy, the degree of financial development within the system of each province in China has been dynamically changing for a long time. Given different degrees of market economy openness, the intensity and efficiency of financial services available to different regions are different. Therefore, differences in financial development lead to heterogeneity in the effect of the digital economy on high-quality agricultural development. Therefore, influenced by the level of financial development, the digital economy is highly likely to have a threshold effect on high-quality agricultural development. The development of the digital economy is based on certain digital infrastructures and information technologies. Regions with low financial development are subject to financing constraints, limited investments in digital infrastructure, limited network coverage, and limited application scope, depth and technical levels. The promotional effect of the digital economy on high-quality agricultural development is not obvious. In areas with high financial development, the expansion of effective financing channels can provide adequate financial support for the research and development of digital information infrastructures and emerging technologies and expand the popularization of digital information technology to fully release the role of the digital economy in high-quality agricultural development, give full play to the dividend effect of the digital economy, and further promote high-quality agricultural development. Therefore, the following hypothesis is proposed.

H2: The promoting effect of the digital economy on high-quality agricultural development is influenced by financial development. The higher the financial development is, the more significant is the promoting effect of the digital economy on high-quality agricultural development.

### The heterogeneous impact of the digital economy on high-quality agricultural development

The global estimation results obtained by the traditional econometric model are only "average" in a certain sense, which cannot reflect spatial heterogeneity, especially the law of changes in the observed samples using spatial locations. However, different regions have different degrees of development of the digital economy and high-quality agriculture due to geographical locations, resource endowments, policy inclinations and economic foundations, which may lead to obvious regional heterogeneity regarding the impact of the digital economy on high-quality agricultural development. That is, given a change in geographical location, the same factors also have different impacts on high-quality agricultural development. Based on the above analysis, the following hypothesis is proposed.

H3: There is heterogeneity in the impact of the digital economy on high-quality agricultural development.

## Research design

### Benchmark regression model

According to the above theoretical analysis, the digital economy has an important impact on high-quality agricultural development. In this paper, a static panel data model is first constructed to test whether financial development moderates the influence of the digital economy on high-quality agricultural development. To weaken the influence of heteroskedasticity on the model, logarithmic processing is used for all variables:

$$ln{ahq}_{it}=\alpha_{0}+\alpha_{1}lnd{igital}_{it}+\beta_{i}X_{it}+\varepsilon_{it}$$ (1)

$$ln{ahq}_{it}=\alpha_{0}+\alpha_{1}lnd{igital}_{it}+\alpha_{2}ln{finance}_{it}+\alpha_{3}lnd{igital}_{it}∙ln{finance}_{it}+\beta_{i}X_{it}+\varepsilon_{it}$$ (2)

Among them, *i* represents the province, *t* represents the year, *ε*_*it*_ is a random perturbation term, *lnahq*_*it*_ represents high-quality agricultural development, *lndigital*_*it*_ represents the digital economy, and *X*_*it*_ is a set of control variables. Formula (1) is the basic econometric model used to simply examine the impact of the digital economy on high-quality agricultural development. To verify the regulatory role of financial development in the digital economy’s influence on high-quality agricultural development, we extend Formula (1) and add the interactive item of digital economy and financial development. In Formula (2), *lnfinance*_*it*_ represents financial development, and *lndigital*_*it*_∙*lnfinance*_*it*_ represents the interactive of digital economy and financial development.

### Panel threshold regression model

If financial development plays a regulating role in the digital economy’s influence on high-quality agricultural development and promotes or restrains this influence, then how can reasonable amounts of financial development be determined? To answer this question, a panel threshold regression model is constructed to investigate the threshold values of the different regulatory roles played by financial development in the impact of the digital economy on high-quality agricultural development:

$$ln{ahq}_{it}=\alpha_{0}+\alpha_{11}lnd{igital}_{it}\times d(q\leq {finance}_{i})+\alpha_{12}lnd{igital}_{it}\times d(q>{finance}_{i})+\beta_{it}X_{it}+\varepsilon_{it}$$ (3)

Compared with Formulas (1) and (2), the meaning of the response variable in Formula (3) has changed. Among them, *d*(*) represents an indicative function, *finance*_*i*_ is a threshold variable, *α*_11_ and *α*_12_ represent separately when *q*≤*finance*_*i*_ and *q*>*finance*_*i*_, the elastic coefficient of the effect of digital economy on the high-quality agricultural development. If the threshold selection is reasonable, the estimates or symbols of *α*_11_ and *α*_12_ should be significantly different. Formula (3) only analyzes the single threshold effect; considering that the analysis process of multiple thresholds is similar to that of single thresholds, no further details are provided. In the empirical analysis section, we conduct multiple threshold verifications and analyses.

### Variable selection and explanation

#### Explained variable

The explanatory variable is high-quality agricultural development (ahq). By constructing the evaluation system of high-quality agricultural development, the index calculated using the entropy method represents comprehensive high-quality agricultural development.

Firstly, establish a high-quality agricultural development evaluation system. The essence of high-quality agricultural development and its fundamental purpose is to "meet the people’s ever-growing needs for a better life", take quality and benefit as the value orientation, and basically follow the five development concepts of "innovation, coordination, green, open and sharing" [37]. Based on the connotation of high-quality agricultural development and the practices of most scholars [38, 39], we select 5 evaluation dimensions, 10 dimension indices and 19 specific indicators to construct an evaluation system for high-quality agricultural development. See Table 1 for details.

**Table 1:** Evaluation system of high-quality agricultural development.

evaluation dimension	dimension index	Specific indicators	Index measurement	Attributes	weight
**Innovation development**	Scientific innovation	Agricultural science input	Agricultural science expenditure/fiscal expenditure (%)	+	0.0799
		Agricultural technician	Number of agricultural technicians	+	0.0643
	Improvement of quality and efficiency	Labor productivity	Primary industrial added value/rural population	+	0.0545
		Land productivity	Primary industrial added value/total sown area of crops	+	0.0827
**Coordinated development**	Industrial coordination	Employment in primary industry	Number of people employed in primary industry	+	0.0778
		Primary industrial added value ratio	Primary industrial added value/GDP	+	0.0434
	Urban‒rural coordination	Urban‒rural income ratio	Per capita disposable income ratio of urban and rural residents	-	0.0194
		Urban‒rural consumption ratio	Per capita disposable consumption ratio of urban and rural residents	-	0.0081
**Green development**	Low carbon production	Pesticide application intensity	Pesticide application/planting area	-	0.0068
		Fertilizer application intensity	Fertilizer application/planting area	-	0.0166
		Rural plastic film application intensity	Rural plastic film application/planting area	-	0.0081
		Forest coverage	Forest area/total land area	+	0.0561
**Open development**	Degree of openness	Degree of agricultural openness	Import and export of agricultural products/GDP	+	0.1206
**Shared development**	Income	Per capita disposable income of rural residents	Per capita disposable income ratio of rural residents	+	0.0509
	Cultural education	Rural labor quality	Average years of schooling for rural residents	+	0.0073
	Hygiene	Number of rural doctors and health workers	Number of rural doctors and health workers	+	0.0944
		Number of village clinics	Number of village clinics	+	0.0935
	Infrastructure	Highway mileage	Highway mileage	+	0.0527
		Degree of production mechanization	Total power of agricultural machinery/planting area	+	0.0630

Secondly, construct the evaluation model of high-quality agricultural development. We followed the methods of Yao W. et al. [36], the entropy method is used to determine the high-quality agricultural development index weights to ensure that these weights are more objective.

Step 1, data standardization. The deviation standardization method is adopted to standardize the original data of all of the indicators. To avoid a zero value, 0.0001 is added on the basis of the standardized formula. We set the number of provinces as k, the number of years as n, and the number of indices as j, so *x*_*ikj*_ is the JTH index value of province k in year i. The positive index data were standardized using Formula (1), and the negative index data were standardized using Formula (2).

Positive index standardization:

$$x_{ikj}^{\prime }=\frac{x_{ikj}-x_{min}}{x_{max}-x_{min}}+0.0001$$ (4)

Negative index standardization:

$$x_{ikj}^{\prime }=\frac{x_{max}-x_{ikj}}{x_{max}-x_{min}}+0.0001$$ (5)

where $x_{ikj}^{\prime }$ is the index data after standardized processing, *x*_*ikj*_ is the original data, *x*_*min*_ represents the minimum value of this index, and *x*_*max*_ represents the maximum value of this index.

Step 2: Determine the index weight:

$$Y_{ikj}=\frac{x_{ikj}^{\prime }}{\sum_{i}\sum_{ikj}^{\prime }}$$ (6)

Step 3: Calculate the entropy value of the JTH index:

$$E_{j}=-r\sum_{i}\sum_{k}Y_{ikj}ln(Y_{ikj}),\;r=ln(in)$$ (7)

Step 4: Calculate the difference coefficient of the JTH index:

$$G_{j}=1-E_{j}$$ (8)

Step 5: Calculate the weight of each index:

$$W_{j}=\frac{G_{j}}{\sum_{j}G_{j}}$$ (9)

Step 6: Calculate the scores of high-quality agricultural development in each province:

$$ahq=\sum_{j}W_{j}x_{ikj}^{\prime }$$ (10)

#### Core explanatory variable

Core explanatory variable is digital economy. We followed the methods of Guo Feng (2020) [40], this paper is based on the past years of the inclusive finance index of prefecture-level cities, data on the number of people in computer services and software, the number of internet broadband users, the number of mobile phone users and telecommunications services revenue. The coefficients of the variation method and the principal component analysis method are used to calculate the scale of the digital economy as a proxy variable for the digital economy.

#### Threshold variable

Threshold variable is financial development (*finance*). This variable is expressed as the proportion of regional financial added value in GDP.

#### Other control variables

Other control variables are as follows: government regulation level (grc), expressed as the proportion of regional financial added value in GDP; industrial structure (is), expressed as the added value of the tertiary industry in the added value of the secondary industry; scientific and technological development (td), expressed as expenditures on science and technology in the general budget for local finances; urbanization level (ul), expressed as the proportion of the total urban population in the total rural population; and degree of openness (open), expressed as the proportion of total agricultural imports and exports in GDP.

### Data sources and descriptive statistics

Based on the scientificity and availability of the data, 31 provinces in China from 2011 to 2020 are selected as research objects. The data in this paper are mainly from the China Rural Statistical Yearbook, China Statistical Yearbook, China Educational Expenditure Statistical Yearbook, and China Science and Technology Yearbook, etc. The specific indicators, data descriptions and statistical descriptions are shown in Table 2.

**Table 2:** Variable definition and descriptive statistics.

Variable name	Variable symbol	Obs	Mean	Std. dev.	Min	Max
**High-quality development of agriculture**	lnahq	310	-1.222	0.321	-2.175	-0.533
**Digital economy**	lndigital	310	5.212	0.677	2.786	6.068
**Financial development**	lnfinance	310	-2.711	0.368	-3.631	-1.628
**Government regulation level**	lngrc	310	-1.356	0.480	-2.124	0.303
**Industrial structure**	lnis	310	0.202	0.381	-0.640	1.657
**Scientific and technological development**	lntd	310	-4.121	0.669	-5.800	-2.695
**Urbanization level**	lnul	310	0.367	0.629	-1.228	2.152
**Degree of openness**	lnopen	310	-4.792	1.139	-7.472	-2.746

## Analysis of empirical results

### Results of measuring high-quality agricultural development

According to the abovementioned evaluation index system and data processing methods, the comprehensive level of high-quality agricultural development in each region can be measured (see Table 3).

**Table 3:** The estimation value of China’s agricultural high-quality development.

area	Province	2011	2012	2013	2014	2015	2016	2017	2018	2019	2020
**Eastern region**	Beijing	0.289	0.322	0.299	0.312	0.304	0.290	0.312	0.329	0.364	0.346
	Tianjin	0.238	0.246	0.247	0.250	0.243	0.226	0.229	0.233	0.255	0.312
	Hebei	0.416	0.416	0.424	0.425	0.415	0.404	0.403	0.398	0.400	0.408
	Liaoning	0.324	0.334	0.344	0.345	0.337	0.328	0.329	0.330	0.334	0.338
	Shanghai	0.216	0.225	0.224	0.229	0.229	0.243	0.259	0.259	0.268	0.283
	Jiangsu	0.327	0.323	0.323	0.324	0.328	0.329	0.336	0.342	0.347	0.350
	Zhejiang	0.333	0.339	0.341	0.342	0.341	0.347	0.352	0.360	0.376	0.375
	Fujian	0.351	0.361	0.370	0.378	0.384	0.388	0.394	0.401	0.411	0.427
	Shandong	0.584	0.576	0.585	0.587	0.553	0.530	0.528	0.524	0.529	0.527
	Guangdong	0.361	0.366	0.378	0.366	0.383	0.399	0.396	0.414	0.427	0.422
	Hainan	0.252	0.256	0.259	0.268	0.267	0.285	0.291	0.296	0.328	0.348
	Mean	0.336	0.342	0.345	0.348	0.344	0.343	0.348	0.353	0.367	0.376
**Central region**	Shanxi	0.229	0.234	0.250	0.253	0.242	0.231	0.229	0.230	0.233	0.245
	jilin	0.258	0.263	0.272	0.266	0.266	0.260	0.259	0.262	0.265	0.281
	Heilongjiang	0.298	0.311	0.336	0.347	0.342	0.341	0.347	0.350	0.360	0.372
	Anhui	0.298	0.305	0.312	0.312	0.313	0.343	0.338	0.343	0.359	0.350
	Jiangxi	0.301	0.308	0.302	0.308	0.310	0.310	0.316	0.316	0.330	0.336
	Henan	0.458	0.439	0.446	0.439	0.432	0.427	0.423	0.420	0.424	0.435
	Hubei	0.316	0.318	0.341	0.343	0.342	0.350	0.358	0.361	0.370	0.365
	Hunan	0.369	0.367	0.372	0.378	0.378	0.382	0.382	0.385	0.399	0.399
	Mean	0.316	0.318	0.329	0.331	0.328	0.330	0.332	0.333	0.343	0.348
**Western region**	Inner Mongolia	0.240	0.240	0.245	0.244	0.246	0.247	0.249	0.254	0.262	0.275
	Guangxi	0.351	0.366	0.366	0.374	0.359	0.355	0.366	0.360	0.359	0.363
	Chongqing	0.197	0.203	0.212	0.214	0.225	0.237	0.235	0.243	0.255	0.270
	Sichuan	0.419	0.420	0.424	0.433	0.435	0.436	0.442	0.446	0.455	0.468
	Guizhou	0.239	0.248	0.252	0.269	0.284	0.289	0.297	0.301	0.303	0.311
	Yunnan	0.336	0.337	0.339	0.346	0.349	0.351	0.354	0.356	0.366	0.387
	Xizang	0.145	0.154	0.161	0.170	0.178	0.180	0.179	0.179	0.185	0.198
	Shaanxi	0.266	0.273	0.270	0.286	0.292	0.293	0.297	0.299	0.302	0.303
	Gansu	0.186	0.195	0.200	0.206	0.213	0.206	0.211	0.211	0.220	0.225
	Qinghai	0.117	0.124	0.134	0.141	0.137	0.141	0.149	0.156	0.166	0.175
	Ningxia	0.114	0.116	0.125	0.133	0.138	0.134	0.141	0.153	0.152	0.161
	Xinjiang	0.214	0.225	0.229	0.227	0.222	0.225	0.223	0.227	0.232	0.243
	Mean	0.235	0.242	0.247	0.254	0.257	0.258	0.262	0.265	0.271	0.282
**China**	Mean value	0.292	0.297	0.303	0.307	0.306	0.307	0.310	0.314	0.324	0.332

Using the comprehensive measurement value of high-quality agricultural development, this measured value in China’s provinces ranges from 0.114 to 0.587 during 2011–2020, and the average measurement value of national high-quality agricultural development ranges from 0.292 to 0.332, showing a steady rising trend overall; however, overall high-quality agricultural development is low. During the study period, the high-quality development of China’s agriculture was stable overall, with a small fluctuation range in the past ten years. After 2017, this development began to increase slightly, and the data analysis results were basically consistent with the actual situation. Shandong, Sichuan, Henan, Hebei and Guangdong ranked as the top five in terms of the mean value of high-quality agricultural development, which was above 0.398. The last five provinces are Xinjiang, Gansu, Tibet, Qinghai and Ningxia, with average of their high-quality agricultural development below 0.229, indicating low-quality agricultural development regions that are far lower than the national average of 0.309. The provinces that rank high in the overall measurement of high-quality agricultural development are closely related to their resource utilization and ecological environment. These regions have abundant total resources and can accelerate the transformation of agriculture, accelerate the transformation of the development mode, constantly optimize the industrial structure, adhere to investments in talent, science and technology, and constantly improve industrial and production efficiency. Some provinces have their own resources and environments that restrict the development of high-quality agriculture. Although some provinces are rich in resources, they are limited by weak production efficiency and insufficient technological innovation.

### Kernel density estimation

**Fig 1:**
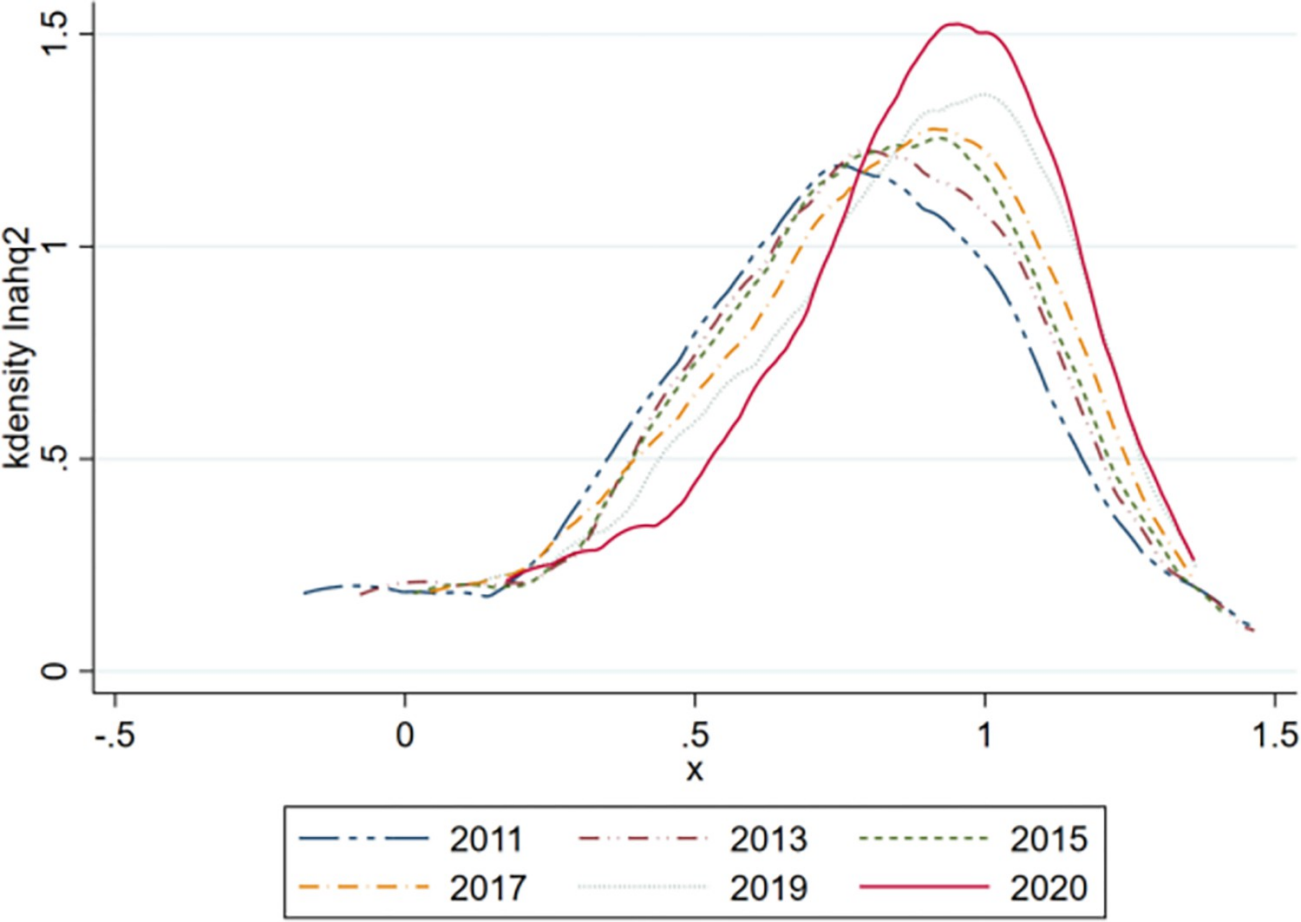
Kernel density estimation of high-quality agricultural development.

As shown in Fig 1, during the study period, the kernel density curve of high-quality agricultural development presented an unimodal distribution, and the kurtosis gradually increased, indicating that the difference in high-quality agricultural development gradually increased, especially in 2020, when the kurtosis was the highest. From the skewness point of view, the curve of the nuclear density of high-quality agricultural development gradually swung from right to left, indicating that high-quality agricultural development increased year by year; however, more areas had low development.

**Fig 2:**
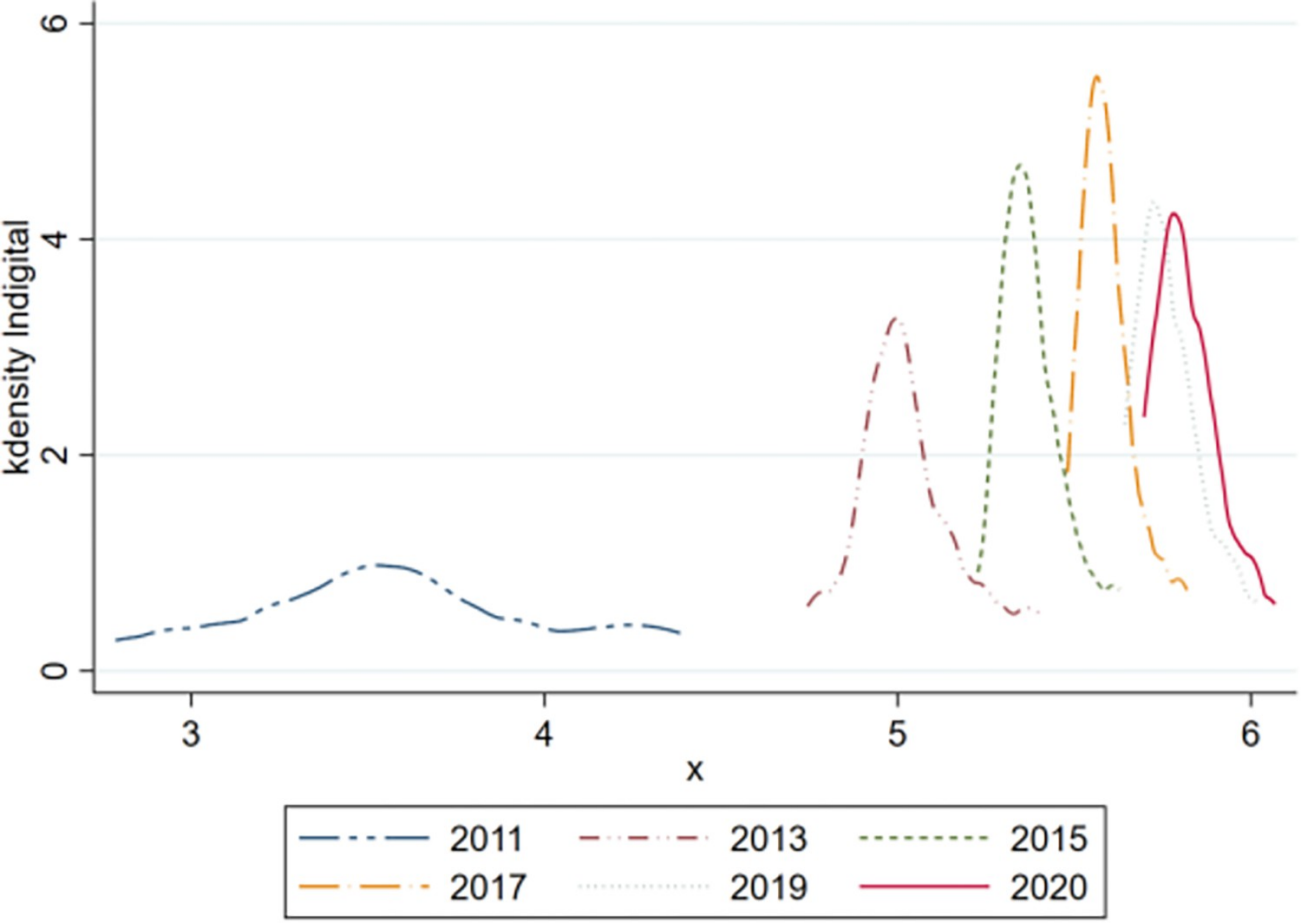
Kernel density estimation of the digital economy.

Fig 2 shows that during the study period, the nuclear density curve of the digital economy also presents an unimodal distribution. From 2011 to 2017, the kurtosis gradually increased. After 2017, the kurtosis gradually decreased, indicating that the difference in the digital economy gradually decreased after 2017. From the perspective of skewness, during the study period, the curve of the nuclear density of the digital economy shifted to the right each year, with a large range of movement that indicates that the development of the digital economy greatly improved and leapfrog development was realized. Meanwhile, there are many regions with a substantial digital economy.

### Baseline regression results and analysis

In this paper, the individual and time two-way fixed effect model is adopted. High-quality agricultural development is used as the explained variable and digital economy is the core explanatory variable for the regression analysis. The regression results are shown in Table 4.

**Table 4:** Regression analysis results of the digital economy on high-quality agricultural development.

	lnahq			
	model_1	model_2	model_3	model_4
**lndigital**	0.0577***		0.319***	0.325***
	(0.00957)		(0.0625)	(0.0590)
**lnfinance**		0.161***	-0.466***	-0.459***
		(0.0298)	(0.115)	(0.0892)
**c.lndigital#c.lnfinance**			0.0879***	0.0965***
			(0.0196)	(0.0190)
**lngrc**				-0.166***
				(0.0561)
**lnis**				-0.0660
				(0.0697)
**lntd**				0.0489**
				(0.0183)
**lnul**				0.123**
				(0.0543)
**lnopen**				0.0408*
				(0.0228)
**Province control**	YES	YES	YES	YES
**Year control**	YES	YES	YES	YES
**_cons**	-1.522***	-0.784***	-2.917***	-2.668***
	(0.0499)	(0.0807)	(0.370)	(0.309)
***N***	310	310	310	310
***R*** ^**2**^	0.378	0.238	0.486	0.626

Standard errors in parentheses

* p < 0.1

** p < 0.05

*** p < 0.01

As shown in Table 4, Model 1 is the benchmark regression of the digital economy on high-quality agricultural development without considering other factors. The regression results show that the digital economy has a significant positive impact on high-quality agricultural development. Model 2 is the baseline regression of financial development to high-quality agricultural development without considering other factors. The results show that financial development has a significant positive impact on high-quality agricultural development. Model 3 reflects the joint influence of the digital economy, financial development and the interaction terms of digital economy and financial development on high-quality agricultural development. The results show that the direction of the effect of the digital economy on high-quality agricultural development has not changed, while the direction of the effect of financial development on high-quality agricultural development has changed from the original positive impact to a negative impact. The interactive terms of the digital economy and financial development have a significant positive impact on high-quality agricultural development. On the basis of Model 3, control variables such as government regulation level, industrial structure, scientific and technological development level, urbanization level and degree of openness are added to Model 4. The results show that the digital economy has a significant positive impact on high-quality agricultural development with an impact coefficient of 0.325, while financial development has a significant negative impact on high-quality agricultural development with an impact coefficient of -0.459. The interaction term of the two has a significant positive effect on high-quality agricultural development, and the influence coefficient is 0.0965.

Among the control variables, the level of government regulations and control has a significant negative impact on high-quality agricultural development, with a coefficient of -0.166, while scientific and technological development, the urbanization level and the degree of opening-up have a significant positive impact on high-quality agricultural development, with coefficients of 0.0489, 0.123 and 0.0408, respectively. The control variable industrial structure has no significant influence on high-quality agricultural development.

Among the control variables, the level of scientific and technological development, the urbanization level and the degree of openness have a significant positive impact on high-quality agricultural development, with coefficients of 0.0489, 0.123 and 0.0408, respectively. The possible explanation is that the higher the level of scientific and technological development, the more conducive it is to breaking through technical barriers in the fields of seed breeding, agricultural machinery and equipment, and processing of agricultural products, as well as accelerating the transformation and application of agricultural scientific and technological achievements, thus promoting high-quality development in agriculture. Urbanization provides market, technical and financial support for high-quality agricultural development, promotes the non-farm transfer of rural labor, optimizes the allocation of agricultural resources, and provides favorable conditions for the transformation and upgrading of agricultural modernization, which promotes high-quality agricultural development. A high degree of openness indicates that it is possible to make full use of foreign agricultural resources to improve agricultural production capacity, enhance the ability to guarantee the supply of domestic agricultural products, and optimize the structure of food production and consumption. Among other control variables, the level of government regulation has a significant negative effect on agricultural high-quality development, with a coefficient of -0.166. This shows that excessive government regulation will make agricultural development deviate from the law of market economy development which is not conducive to the high-quality agricultural development. The industrial structure has a negative effect on agricultural high-quality development, but does not pass the significance test. The results of Models 3 and 4 show that financial development has a significant positive or negative regulatory effect in the process of the digital economy affecting high-quality agricultural development. The specific effect is explained in the threshold effect analysis below.

### Endogenous exploration

The results of the benchmark regression indicate that the digital economy has a significant driving effect on high-quality agricultural development, but this result may be subject to endogeneity bias due to bidirectional causality and omitted variables. Therefore, this paper adopts the instrumental variable method to test the benchmark regression results. Considering that the degree of terrain undulation affects the construction of digital infrastructure, which in turn affects the speed of digital transformation. Therefore, this paper draws on the study of Guo JG, et al. [41], and selects the interaction term between provincial terrain undulation degree and time t as well as the lag one period of the dependent variable as instrumental variables for endogeneity test.

The validity of external instrumental variables must be tested when using the method of instrumental variables to perform two-stage least square regression. Table 5 reports the results of a series of tests on the instrumental variables.

**Table 5:** Regression results of instrumental variables.

model	lnahq	
	(1)	(2)
**lndigital**	0.0696***	0.0321***
	(0.0253)	(0.0215)
**Control variable**		YES
**Province control**	YES	YES
**Year control**	YES	YES
**Anderson canon.corr.LM**	473.92	183.926
**statistic**	[0.0000]	[0.0000]
**Cragg-Donald Wald F**	256.986	199.704
**statistic**	{16.38}	{16.38}
**N**	310	310

Standard errors in parentheses. The p-value of the test statistic is in brackets. The critical values of the Stock-Yogo test at the 10% and 15% levels are in braces.

* p < 0.1

** p < 0.05

*** p < 0.01

Firstly, column (1) is an instrumental variable regression with high quality agricultural development as the dependent variable without adding other control variables. The results show that the unidentifiable tests of Anderson canon corr.LM all reject the original hypothesis at a significant level of 1%. This indicates that the exogenous instrumental variables selected are correlated and identifiable with the endogenous explanatory variables. The Cragg-Donald Wald F statistic value of weak instrumental variable test was 256.986, both of which were significantly greater than the critical value of 16.38 at the significance level of 10%. This shows that the null hypothesis of weak instrumental variables is rejected.

Secondly, column (2) is an instrumental variable regression of high-quality agricultural development as the dependent variable with the addition of other control variables. The results show that the unidentifiable tests of Anderson canon corr.LM all reject the original hypothesis at a significant level of 1%. This indicates that the selected exogenous instrumental variables are correlated with the endogenous explanatory variables and can be identified. The Cragg-Donald Wald F statistic value of weak instrumental variable test was 199.704, both of which were significantly greater than the critical value of 16.38 at the 10% significance level. This means that the null hypothesis of weak instrumental variables is rejected.

Finally, in the regression results, the coefficient size, direction and significance of the core explanatory variables and each control variable are basically consistent with the benchmark regression. Therefore, there is no endogeneity problem in the regression results.

### Robustness test

We tested the robustness of the model by replacing, increasing, and reducing the control variables. The results are shown in Table 6.

**Table 6:** Robustness test.

	lnahq			
	model_4	model_5	model_6	model_7
**lndigital**	0.325***	0.331***	0.318***	0.305***
	(0.0590)	(0.0529)	(0.0578)	(0.0527)
**lnfinance**	-0.459***	-0.490***	-0.451***	-0.455***
	(0.0892)	(0.0928)	(0.0866)	(0.0879)
**c.lndigital#c.lnfinance**	0.0965***	0.0984***	0.0945***	0.0892***
	(0.0190)	(0.0166)	(0.0186)	(0.0167)
**lngrc**	-0.166***	-0.174**	-0.161***	-0.198***
	(0.0561)	(0.0676)	(0.0551)	(0.0631)
**lnis**	-0.0660	-0.0451	-0.0644	
	(0.0697)	(0.0612)	(0.0700)	
**lntd**	0.0489**		0.0501**	0.0524**
	(0.0183)		(0.0188)	(0.0197)
**lnul**	0.123**	0.159***	0.121**	0.0888**
	(0.0543)	(0.0551)	(0.0559)	(0.0431)
**lnopen**	0.0408*	0.0601**	0.0420*	0.0328
	(0.0228)	(0.0227)	(0.0231)	(0.0258)
**lnatd**		0.142**		
		(0.0564)		
**lnre**			0.0852	
			(0.139)	
**Province control**	YES	YES	YES	YES
**Year control**	YES	YES	YES	YES
**_cons**	-2.668***	-1.822***	-2.797***	-2.722***
	(0.309)	(0.496)	(0.397)	(0.325)
***N***	310	310	310	310
***R*** ^**2**^	0.626	0.635	0.627	0.618

Standard errors in parentheses

* p < 0.1

** p < 0.05

*** p < 0.01

In Model 5, the development of agricultural science and technology, “lnatd” (logarithm of the proportion of agricultural researchers in the total population of the region), is used as the proxy variable for the development of science and technology, replacing “lntd” in the original Model 4. On the basis of the original Model 4, Model 6 adds the control variable of rural education investment, “lnre” (logarithm of rural per capita years of schooling). In Model 7, the control variable “lnis” is eliminated on the basis of the original Model 4. The regression results in Table 6 show that in the transformed Models 5, 6 and 7, the interaction terms of digital economy, financial development, and digital economy and financial development do no significantly change the direction and significance of the effect on high-quality agricultural development, indicating that the original model is robust.

### Financial development threshold estimation

According to the results of the benchmark regression mentioned above, financial development plays a certain regulating role in the process of the digital economy’s influence on high-quality agricultural development. Therefore, the panel threshold model is adopted for testing. In this paper, STATA 17.0 software and the bootstrap method were used to test the threshold effect with financial development as the threshold variable by repeated the bootstrap sampling 300 times. The results are shown in Table 7.

**Table 7:** Threshold effect self-sampling test.

Threshold variable	Explained variable	Threshold	F	P	BS frequency
**Financial development**	High-quality development of agriculture	Single	64.29	0.0033	300
		Double	48.92	0.0067	300
		Triple	15.65	0.6600	300

The results in Table 7 show that with financial development as the threshold variable, the single and double threshold test is passed at the 1% significance level, while the triple threshold fails the significance test. This indicates that in the process of the digital economy’s influence on agricultural quality, financial development has a double threshold. After the threshold effect self-sampling test, the threshold value of the panel threshold model is estimated and tested, and the results are shown in Table 8.

**Table 8:** Results of threshold value estimation.

Threshold	Estimated value	95% conf.interval
**First Threshold**	0.0711	[0.0706,0.0714]
**Second Threshold**	0.1391	[0.1376,0.1409]

**Fig 3:**
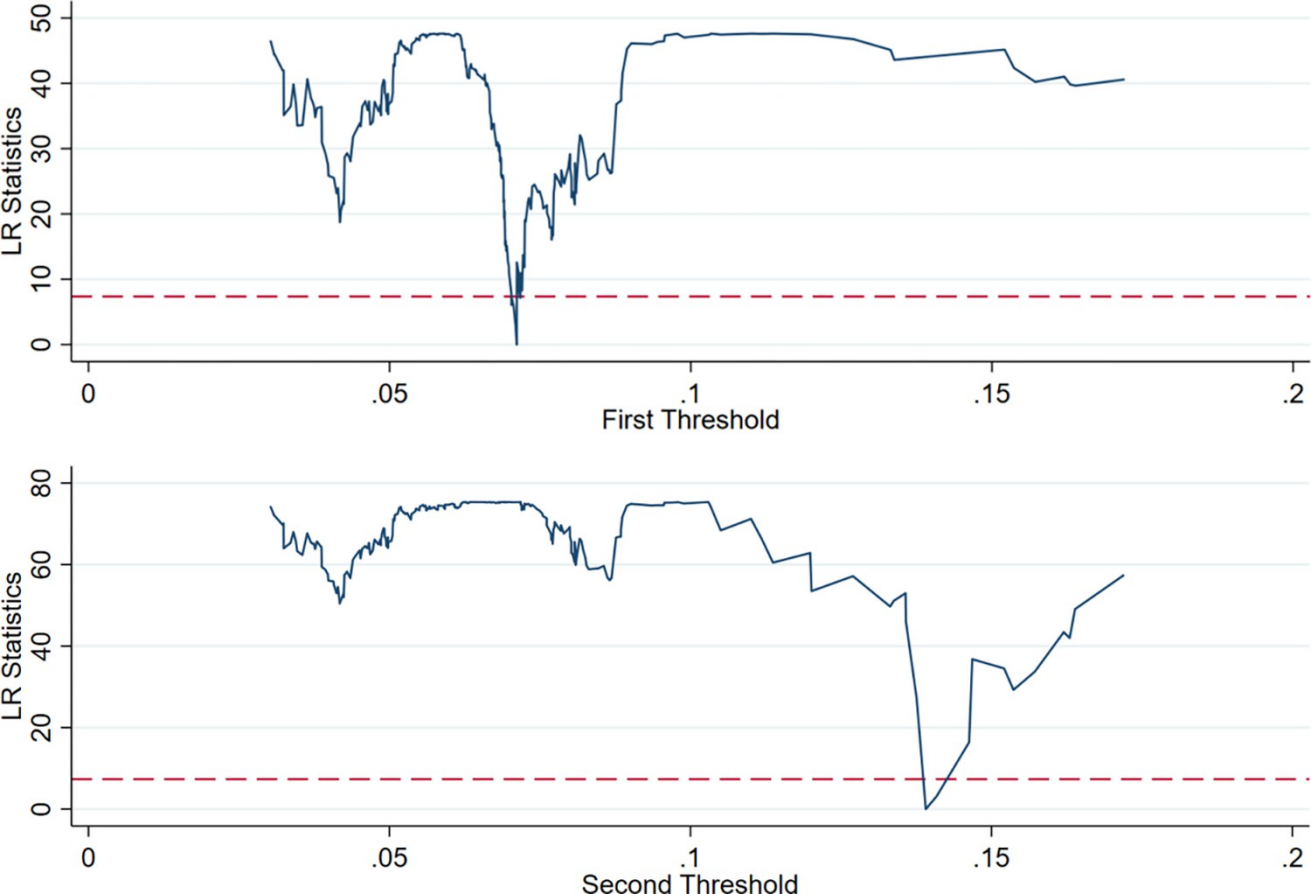
Double threshold estimation results and confidence intervals.

According to the estimation method proposed by Hansen, the threshold value is the corresponding γ value when the likelihood ratio statistic LR tends to 0. Therefore, the LR graph (Fig 3) with the estimated value of the relative response threshold under the 95% confidence interval is drawn to intuitively show the construction process of the estimated value and confidence interval of the two thresholds of financial development. In the LR graph, below the dotted line is the threshold interval corresponding to the critical value of 7.35 when the LR value is less than the 5% significance level. According to Table 8 and Fig 3, the distribution of the estimated double threshold of financial development is 0.0711 and 0.1391, and the corresponding confidence intervals are [0.0707–0.0714] and [0.1376–0.1409]. Therefore, the financial development of China’s 31 provinces (autonomous regions and municipalities) can be divided into three levels during the study period: low financial development (finance<0.0711); medium financial development (0.0711≤finance≤0.1391); and high financial development (finance>0.1391). The impact of the digital economy on high-quality agricultural development in different types of regions is shown in Table 9.

**Table 9:** Estimation results of double threshold parameters of financial development.

	variable	lnahq		
		Coefficient	T	95%conf.interval
**Core explanatory variable**	finance<0.0711	0.0245***	3.55	[0.011,0.382]
**lndigital**	0.0711≤finance≤0.1391	0.0342***	4.87	[0.020,0.048]
	finance>0.1391	0.0669***	9.00	[0.052,0.082]
	Control variable	Control	-	-
	_cons	-1.290***	-13.61	[-1.477,-1.103]
	N	310	310	310
	*R* ^2^	0.653	-	-

Standard errors in parentheses

* p < 0.1

** p < 0.05

*** p < 0.01

The estimation results of the double threshold parameters of financial development show that during the study period, China’s digital economy has different impacts on agricultural quality given different financial development levels (see Table 9). According to the regression results, at low financial development (finance<0.0711), the digital economy has a positive impact on high-quality agricultural development, and the coefficient is 0.0246, which passes the test at the 1% significance level. At medium financial development (0.0711≤finance≤0.1391), the digital economy has a positive impact on high-quality agricultural development at the 1% significance level, indicating that the digital economy has a further increasing role in promoting high-quality agricultural development within this interval. In the region with high financial development (finance>0.1391), the digital economy has a positive impact on high-quality agricultural development at the 1% significance level, indicating that within this region, the effect of the digital economy on high-quality agricultural development increases exponentially and has a significant promoting effect. It can be seen that as financial development improves, the impact of the digital economy on high-quality agricultural development presents a role path of "promoting, promoting and doubling".

The main reason is that when the level of financial development is low, the financial support for the construction of digital infrastructure in rural areas will be weakened, and the application scope of smart agricultural technologies such as agricultural robots, numerical control spraying, and intelligent detection will be limited, which is not conducive to the transformation and upgrading of agricultural production mode, so that the digital economy has a limited role in promoting the high-quality agricultural development. When the financial development reaches a certain level, it can provide a relaxed financial environment for the development of the digital economy, and provide sufficient funds for the improvement of rural digital infrastructure, which is conducive to the promotion of efficient, precise and intelligent agricultural technology, and better improve the level of agricultural automation and production efficiency. Thus, the enabling effect of digital economy on the high-quality agricultural development has been greatly improved. Therefore, the threshold effect shows that when financial development crosses the threshold, the driving effect of the digital economy on high-quality agricultural development increases substantially, which further verifies that hypothesis 2 is valid.

### Heterogeneity analysis

#### Regional heterogeneity analysis

Due to the differences in the development of the digital economy and agriculture in different regions of China, the impact of the digital economy on high-quality agricultural development in different regions may be different. To test whether the digital economy can only promote high-quality agricultural development in some regions, in this paper, an individual and time bidirectional fixed effect model was adopted to test and analyze the heterogeneity of the impact of the digital economy on high-quality agricultural development in the eastern, central and western regions. The regression results are shown in Table 10. As shown in Table 10, the digital economy in eastern China can significantly promote high-quality agricultural development, with an impact coefficient of 0.0752, while the digital economy in central and western China can significantly promote high-quality agricultural development, with impact coefficients of 0.0123 and 0.0213, respectively. However, both fail to pass the significance test, indicating significant differences in the effect of the digital economy on high-quality agricultural development in different regions, with the eastern region having the largest effect, and the central and western regions having a smaller effect. This regional difference is mainly due to the higher level of financial development, superior market environment, higher level of digital literacy among farmers, and well-developed and improved transportation infrastructure in the eastern region, all of which are favorable conditions to support high-quality development of agriculture in the east. Compared with the eastern region, the digital economy plays a less important role in enhancing the high-quality development of agriculture in the central and western regions. The possible reasons are that the current cost of digital transformation of agricultural production in the central and western regions is still high for small farmers, the popularity of the application of digital devices such as agricultural robots, biosensors, and the Internet of Things (IoT) in agricultural production is not high, and there are still technical barriers to the application of agricultural big data and Internet platforms by farmers. Therefore, the marginal contribution of the digital economy to high-quality agricultural development in the central and western regions is relatively small.

**Table 10:** Results of the heterogeneity test.

	Eastern region	Central region	Western region	Major grain-producing area	Major grain-selling area	Balance of production and sales area
	model_8	model_9	model_10	model_11	model_12	model_13
**lndigital**	0.0752***	0.0123	0.0213	0.249*	0.269***	0.0408
	(0.0178)	(0.0152)	(0.0134)	(0.119)	(0.0651)	(0.139)
**lnfinance**	-0.155*	0.0582	-0.00660	-0.355*	-0.385***	0.0967
	(0.0711)	(0.0842)	(0.0321)	(0.173)	(0.116)	(0.251)
**lngrc**	-0.137*	-0.131	-0.0800	-0.0898	-0.0586	-0.106
	(0.0674)	(0.114)	(0.0990)	(0.0933)	(0.0574)	(0.0600)
**lnis**	0.220**	0.129	-0.158**	0.122	-0.159***	0.124
	(0.0830)	(0.0928)	(0.0549)	(0.0851)	(0.0424)	(0.110)
**lntd**	0.107***	0.0556	0.0623**	0.0625**	0.0645**	0.0578
	(0.0327)	(0.0378)	(0.0253)	(0.0281)	(0.0273)	(0.0338)
**lnul**	-0.0411	-0.156	0.321***	-0.167	0.249***	0.278**
	(0.0940)	(0.117)	(0.0657)	(0.124)	(0.0475)	(0.107)
**lnopen**	0.216**	0.0125	0.00886	0.0783*	0.0235	0.235***
	(0.0695)	(0.0118)	(0.0141)	(0.0422)	(0.0174)	(0.0515)
**Province control**	YES	YES	YES	YES	YES	YES
**Year control**	YES	YES	YES	YES	YES	YES
**_cons**	-0.957	-0.883*	-1.260***	-1.788***	-2.385***	-0.603
	(0.552)	(0.384)	(0.335)	(0.395)	(0.494)	(0.773)
***N***	110	80	120	130	70	110
***R*** ^**2**^	0.594	0.521	0.834	0.449	0.845	0.840

Standard errors in parentheses

* *p* < 0.1

** *p* < 0.05

*** *p* < 0.01

#### Resource endowment heterogeneity analysis

In order to examine the impact of the digital economy on the high-quality agricultural development under different resource endowments, this paper refers to China’s 2001 grain circulation system reform, based on the characteristics of grain production and marketing in various regions as well as the differences in resource endowments, the sample is divided into three major agricultural functional areas: the major grain-producing areas, the major grain-selling areas, and the balance of production and sales areas. Among them, the major grain-producing areas include Liaoning, Jilin, Heilongjiang, Henan, Hebei, Hunan, Hubei, Jiangxi, Anhui, Sichuan, Jiangsu, Inner Mongolia and Shandong. The major grain-selling areas include Beijing, Shanghai, Tianjin, Zhejiang, Guangdong, Fujian, Hainan. The balance of production and sales areas include Guangxi, Chongqing, Yunnan, Guizhou, Shanxi, Shaanxi, Qinghai, Gansu, Ningxia and Xinjiang.

Table 10 shows that the digital economy is favorable to the high-quality agricultural development in the major grain-producing areas and the major grain-selling areas. In addition, the positive impact effect of the digital economy in the major grain-selling area is greater. However, the positive driving effect of the digital economy in the balance of production and sales areas is not significant. The economic logic may be that the major grain-selling areas are mostly economically developed regions with more advanced digital infrastructure. By relying on online platforms and the extensive use of digital technology, the marketing mode of agricultural products has become more diversified. And it has improved the circulation efficiency and promoted the quality and efficiency of agriculture. In major grain-producing areas, the development of digital economy is conducive to the construction of big data centers for the agricultural industry chain, which can provide agricultural producers and operators with information related to agricultural operation and management, and improve the efficiency of factor allocation.

## Conclusions and suggestions

Based on the measurement of the high-quality agricultural development level of 31 provinces in China during 2011–2020, we empirically test the impact of the digital economy on high-quality agricultural development. The above research shows the following. (1) During the study period, the high-quality development level of China’s agriculture showed a steady upward trend; however, the regional differences were significant, with eastern China’s development being higher than that of central and western China. (2) The digital economy can promote high-quality agricultural development. (3) The digital economy has a double threshold effect in the process of affecting high-quality agricultural development, which depends on financial development. When the threshold is exceeded, the digital economy has a more significant promoting effect on high-quality agricultural development. (4) The impact of the digital economy on high-quality agricultural development is heterogeneous. From the perspective of different regions, the impact effect is greatest in the eastern region, while the effect is smaller in the central and western regions. From different resource endowments, the positive impact effect is greatest in the major grain-selling areas, followed by the major grain producing areas, but the positive digital economy driving effect is not significant in the balance of production and sales areas. Accordingly, we make the following policy recommendations.

First, given the reality that the digital economy promotes high-quality agricultural development, the government should increase investments in and support for digital technology and promote the integration of the digital economy and agriculture. Specifically, the government can increase funding for digital technology research and development in universities and research institutes through project entrustment and in other ways, strengthen the transformation and application of digital technology research and development achievements in agricultural development, and constantly improve the degree of integration of the digital economy and high-quality agricultural development.

Second, efforts should be made to promote the development of digital inclusive finance in areas with financial development below the threshold. Digital technology can help rural finance expand the coverage of digital inclusive financial services in rural areas, accelerate the establishment of a rural digital financial credit system, optimize agricultural digital financial insurance services, enable the use of digital platforms to establish the internal connection between agricultural insurance and agricultural credit, and enable the formation of an organic combination of online and offline digital agricultural finance development modes through the service model of “run more with data, run less with farmers", effectively promoting high-quality agricultural development.

Third, different regions should develop implementation plans to promote high-quality agricultural development through a differentiated digital economy. On the one hand, the eastern region should make full use of its favorable conditions in the market environment, digital infrastructure, agricultural science and technology research and development, rural households’ digital literacy and other aspects, accelerate the development and promotion of digital technology application scenarios in the development of the agricultural industry, promote digital technology innovation in agricultural science and technology and transform scientific research achievements, and increase support for rural households’ innovation and entrepreneurship, as well as play a leading or exemplary role for the central and western regions. On the other hand, the central and western regions should accelerate efforts to strengthen the weak links in the basic elements of digital rural construction, strengthen the construction of internet, mobile broadband, 5G and other digital infrastructure in rural areas, and focus on building and creating a sound digital ecological environment. Through these actions, the training of rural digital personnel and the growth of leaders for the development of digital agriculture and the gradual closing of the digital divide among the eastern, central and western regions can be accelerated.

## Supporting information

S1 Data (XLSX)
